# Ventilatory response during intentional early rehabilitation in patients with mechanical ventilation

**DOI:** 10.1186/cc14343

**Published:** 2015-03-16

**Authors:** K Obata, N Shiba, T Takahashi, S Ichiba, Y Ujike

**Affiliations:** 1 Japanese Red Cross Okayama Hospital, Okayama, Japan; 2 Okayama University Graduate School of Medicine, Okayama, Japan; 3 Tokyo University of Technology, Tokyo, Japan

## Introduction

Intentional early rehabilitation with mechanical ventilation in ICUs is performed in clinical settings. However, strict indexes for safe rehabilitation have not been fully elucidated. The purpose of this study is to analyze ventilator response (VR) between healthy volunteers and patients with mechanical ventilation.

## Methods

Sixteen healthy volunteers (Control group) and 13 patients on mechanical ventilation (MV group) were enrolled in this study. Both groups were positioned in a variety of postures (baseline in a supine position, settled back 30°, settled back 60°, and sitting position) and measured with an indirect calorimeter. The instantaneous energy expenditure (EE), tidal volume (TV), respiratory rate (RR) and minute expiratory volume (VE) were non-invasively measured in each posture. The VE was indexed by body weight and the EE was also indexed by the basal energy expenditure (BEE) estimated by the Harris-Benedict formula. VR was defined as the slope in the indexed VE-indexed EE plot with an assumption of those relationship in the linear manner. We examined the correlation between indexed EE and indexed VE in both groups, and the differences of the maximal indexed EE, the maximal indexed MV, and the others between both groups were investigated using the unpaired *t *test. For all the data, significance was accepted at values of *P *< 0.05.

## Results

There was a significant correlation between the indexed EE and indexed VE in both groups (*R *= 0.51, *P *< 0.0001 in the control group; *R *= 0.63, *P *< 0.0001 in the MV group). The VR was significantly suppressed in the MV group compared with the control group (0.041 ± 0.003/minute/ BEE vs. 0.069 ± 0.003/minute/BEE, *P *= 0.012; respectively). Although the indexed VE was comparable in the MV and control groups (0.19 ± 0.07 l/ kg vs. 0.17 ± 0.04 l/kg, *P *= 0.23; respectively), the indexed EE was shifted to a higher range in the MV group than in the control group (maximal indexed EE; 2.26 ± 0.68 vs. 1.74 ± 0.20, *P *= 0.008; respectively). The TV was smaller (maximal TV; 985 ± 592 ml vs. 1,410 ± 299 ml, *P *= 0.018; respectively) and the RR was more frequent (maximal RR; 30 ± 8/minute vs. 16 ± 4/minute, *P *< 0.0001; respectively) in the MV group than in the control group.

## Conclusion

The VR to external stress with mechanical ventilation is more suppressed than in healthy volunteers. The VE in the mechanical ventilation was earned by a higher RR rather than by increased TV. Careful monitoring of VE or RR would be beneficial in early rehabilitation with mechanical ventilation.

